# Differential Suppressive Effects of Testosterone on Immune Function in Fresh Water Snake, *Natrix piscator*: An *In Vitro* Study

**DOI:** 10.1371/journal.pone.0104431

**Published:** 2014-08-07

**Authors:** Manish Kumar Tripathi, Ramesh Singh

**Affiliations:** Department of Zoology, Udai Pratap Autonomous College, Varanasi, Uttar Pradesh, India; University of Missouri-Kansas City, United States of America

## Abstract

Reptiles represent the crucial phylogenetic group as they were the ancestors of both birds and mammals hence very important to study. The objectives of the present study were to investigate the potential roles of testosterone in the innate immune responses and splenic lymphocyte proliferation in fresh water snake, *Natrix piscator*. Animals were mildly anesthetized and spleens were taken out to study the splenic macrophage phagocytosis, super oxide production and nitrite release using *in vitro* testosterone. Splenic lymphocytes were isolated by density gradient centrifugation and were studied for mitogen induced proliferation in presence of in vitro testosterone. Testosterone suppressed the phagocytosis and nitrite release in a concentration dependent manner. Biphasic suppressive effect of testosterone was observed in superoxide production as judged by reduction of nitroblue tetrazolium salt where salt reduction was suppressed at lower and higher concentrations of testosterone. Mitogen induced splenic lymphocyte proliferation was also suppressed by testosterone. By suppressing immune responses, testosterone may, therefore, act as a physiological mechanism regulating the relative amount of energy invested into either reproductive effort or immunocompetence.

## Introduction

The immune system is a coordinated unit consisting of variety of cellular and humoral components that responds to foreign pathogens/factors, with the goal being to eliminate them and return to steady state that existed prior to detection of the same. Sexual dimorphism in immune functions is illustrated in various vertebrate taxa including human [1]–[6]. Field studies in mammals and birds have illustrated that the prevalence (i.e. proportions of the individual infected) and intensity (i.e. severity of the infection) of parasitic infections are often higher in male than female [7], [8]. Laboratory studies have demonstrated that males are more susceptible to infection than females in rodents, and this difference is related to the gonadal steroids [9]–[13]. Males generally exhibit lower immune responses than the female conspecifics [2], [8]. Specifically, the humoral immune responses (i.e., antibody production by B-cells) are typically elevated in females, as compared with males. Females of various species display higher IgM, IgG and IgA concentrations than males and are also better able to mount both primary and secondary antibody response to antigenic challenge than males [1], [14], [15].

Use of sex steroids or its receptor-antagonists or gonadectomy in animal models for studying the pathophysiology and immunoendocrinology has provided direct evidence for the role of sex steroids in immune modulation [16], [17]. Experimental evidences suggest that physiological concentrations of female sex hormones stimulate; while those of male sex hormones suppress the immune responses [18], [19]. Macrophages are considered one of the first surveillance cells of the immune system to encounter nonself elements [20], [21] and to remove them directly by phagocytosis or indirectly by cytotoxicity through release of reactive oxygen/nitrogen intermediates (ROI/RNI) [22], cytokines, such as Interlekin-1 (IL–1) [23], and tumor necrosis factor–α (TNF–α) [24]. The modulation of macrophage function by sex steroids has been the subject of intense investigation in mammals [25]–[28]. The *in vivo* and *in vitro* studies have demonstrated the differential effects of male and female sex steroids on macrophage activities. Physiological concentration of estrogen has been reported to stimulate phagocytosis, proliferative capacity, and tumor cell cytostasis of macrophages [25], [29]; whereas that of androgen, to suppress the cytotoxic activity of macrophages [28], [30]. Dose dependent suppressive effect of dihydrotestosterone (DHT) has been demonstrated on nitrite release by murine peritoneal macrophages [28]. However, Flynn (1986) [31] demonstrated that *in vivo* treatment of testosterone had no change of IL-1 production from murine peritoneal macrophages.

Reptiles, being phylogenetically important, become a pivotal group to study in order to provide significant insights into both the evolution and functioning of the immune system. Only two studies are available in reptiles by Mondal and Rai (1999,2001) [3], [32]. They have reported that sex steroids modulate phagocytosis and cytotoxic responses of splenic macrophages in the wall lizard. There is no report on this aspect in an ophidian, which have lacertilian lineage. Reptiles are generally long-lived, with an extended period of growth and maturation early in life. However, reptiles are unable to internally regulate their body temperature, and undergo strong seasonal change in behavior associated with environmental temperatures. Collectively, these characteristics may have profound effects on how reptiles partition energy resources to self-maintenance activities, including immune function. Hence, this study was performed in the fresh water snake, which is the first report of its kind. In this study, effects of *in vitro* testosterone on phagocytosis and cytotoxic responses of splenic macrophage as well as on mitogen induced splenic lymphocyte proliferation were studied.

## Materials and Methods

### Animals

Freshwater snakes, weighing 80–120g, were obtained from a local supplier who collected these animals in the suburbs of Varanasi (28^0^ 18′N; 83^0^ 01′E). As sexual dimorphism is evident, only males were used in this study during April and May, when animals were reproductively inactive [33]. Animals were brought to the unconditioned laboratory experiencing natural ambient environmental conditions (Max. Temp. 36–39 °C, Min. Temp.24–25 °C; photoperiod 12.5–13.20 h; Relative humidity 40–45%). Animals were housed in cages (size 50x30x30 cm). Each cage had wooden floor and frame with wire net sides, one side being window. Each cage had an earthen bowl (4 L capacity) filled with water to accommodate 4–5 snakes. Snakes were fed on small fishes once a week. Cages were cleaned, and bowl water was changed next day following feeding. Animals were acclimated to the laboratory conditions for two weeks, and experiments were performed. This study, involving use of snakes, was approved by Institutional Ethics Committee, Udai Pratap Autonomous College, Varanasi, India. The guidelines of the committee for the purpose of control and supervision of experiment on animals (CPCSEA), Ministry of Statistics & Programme Implementation, Government of India, were strictly followed in maintenance and sacrifice of animals.

### Chemicals

MTT [3-(4, 5-dimethylthiozol-2-yl)-2, 5 diphenyl tetrazolium bromide], NBT (Nitroblue Tetrazolium salt), mitogens [PHA (Phytohemagglutinin), ConA (Concanavalin A) and LPS (Lipopolysaccharide)] and testosterone were purchased from Sigma Chemicals. Culture medium (RPMI-1640), lymphocyte separation medium (HiSep), L-glutamine, gentamycin, fetal bovine serum (FBS), dimethyl sulfoxide (DMSO), and other chemicals were purchased from Himedia Laboratories Pvt. Ltd. (India). The culture medium was supplemented with 1 µl ml^–1^ gentamycin, 10 µl ml^–1^ of 200 mM L-glutamine, 10 µl ml^–1^ anti-anti (Gibco) and 5% FBS and referred to as complete culture medium.

### Preparation of macrophage monolayer

Snakes were given mild anesthesia of nembutal (sodium pentobarbital), spleen was taken out and then animals were sacrificed with nembutal overdose (100 mg/kg body weight). Care was taken to strictly follow the guidelines of Institutional Ethics Committee for experimentation on animals. Preparation of splenic macrophage monolayer and phagocytic assay were performed following the method of Mondal and Rai (1999,2001) [3], [32]. Briefly, spleen was excised under aseptic conditions and immediately macerated through a nylon strainer of pore size <100 µm into complete culture medium (2 ml per spleen) to get single cell suspension under a sterile laminar flow hood. Thereafter, the suspension was centrifuged at 600×g for 15 min and red blood corpuscles (RBCs) in pellet were lysed by hemolysate buffer. Cell viability, which exceeded 95%, was checked light microscopically through trypan blue exclusion test. Splenic cell suspension (200 µl) was flooded onto individual pre-washed and sterilized glass slides. Phagocytic macrophages were allowed to adhere by incubating the slides at 25°C in humidified CO_2_ atmosphere for 90 min. Non adherent cells were washed off with 0.2 M phosphate buffer saline (PBS; pH 7.2). The splenic macrophage monolayer was prepared in duplicate from each spleen. In the adherent cell population, more than 90% of the cells were macrophages as judged by their morphology.

### Phagocytic assay

For phagocytic assay, the yeast cells were used as target. The yeast cell suspension was prepared by mixing 20 mg of commercial baker’s yeast (*Saccharomyces cerevisae*) in 10 ml of 0.2 M PBS. The suspension was kept at 80°C for 15 min. The cells were washed thrice in PBS and finally suspended in complete culture medium to get a concentration of 1x10^8^ cells ml^–1^. The prepared macrophage monolayer, as above, was flooded with yeast cell suspension, and phagocytosis was allowed to proceed. Following 90 min incubation at 25°C in humidified CO_2_ atmosphere, the slides were rinsed thrice in PBS, fixed in methanol, stained with Giemsa, and examined under oil immersion. For each slide, a total of 100 macrophages were examined randomly without any predetermined sequence. The phagocytic index was determined by calculating the average number of yeast cells engulfed by single macrophage. The percent phagocytosis was calculated by counting the number of macrophages showing phagocytosis per 100 macrophages observed.

### Dose-related effects of *in vitro* testosterone on phagocytosis

Splenic macrophages monolayer prepared on slides were exposed to five different concentrations (0.1, 1, 10, 50 and 100 ng ml^–1^) of testosterone. After 4 hour of incubation, the monolayers were washed three times to remove testosterone and phagocytosis was allowed to proceed.

### NBT assay

NBT assay was performed following the methods of Berger and Slapnickova (2003) [34]. Splenocytes were counted and adjusted to 2x10^6^ cells ml^–1^ in complete culture medium. Cell viability, checked through trypan blue exclusion test, exceeded 95%. Fifty microlitres of cell suspension (1x10^5^ cells) was seeded with 50 µl of different concentrations of testosterone (final concentration of 10, 100 and 1000 ng ml^–1^) in wells of culture plate (96 well) and incubated for four hours in humidified CO_2_ atmosphere at 25°C, following which 50 µl of culture medium containing NBT (1 mg ml^–1^) was mixed. Well with Culture medium (150 ml) without cells served as blank. All assays were performed in triplicates for each spleen. Plate was then incubated for 2 h, centrifuged at 700×g, washed with PBS and fixed in 70% methanol. The formazan crystals, present inside the cells, were dissolved by mixing 20 µl of 0.1% triton X-100, 120 µl KOH (2 M) and 140 µl DMSO. Optical density was measured at 620 nm with the help of ELISA plate reader (Thermo Multiscan). Following blank subtraction, triplicates were averaged.

### Nitrite assay

Nitrite content was measured by the method of Ding et al. (1988) [22]. Briefly, 100 µl of splenocytes (1x10^6^ cells ml^–1^) was added in wells of culture plate (96 well). After two hours of incubation in CO_2_ atmosphere at 25°C, cells were washed with PBS. Fifty microlitres of fresh culture medium and 50 µl of different testosterone concentrations (final concentration of 10, 100 and 1000 ng ml^−1^) were added to each well, and then plates were incubated at for 24 h. Following incubation, plates were then centrifuged at 200×g, and supernatant was collected. Equal volume (50 µl) of supernatant (macrophage conditioned medium) and Griess reagent (1% sulfanilamide in 3N HCl and 0.1% naphthylenediamine dihydrochloride in distilled water) are mixed, and the optical density of solution was measured at 540nm with the help of ELISA plate reader (Thermo Multiscan). Culture medium without cells served as blank. All assays were performed in triplicates. Following blank subtraction, the triplicates were averaged. The standard curve of sodium nitrite was used to calculate the amount of nitrite in the conditioned medium. The concentration of nitrite is expressed in micromole (µM).

### Splenic lymphocyte proliferation assay

Splenic single cell suspension prepared as above was treated with hemolysate buffer (0.15 M NH_4_Cl, 10 mM KHCO_3_, 0.1 mM Na_2_EDTA, pH 7.2), washed with 0.2 M PBS (pH 7.2) thrice and resuspended in complete culture medium. Splenic lymphocytes were isolated by density gradient centrifugation using HiSep (Density 1.077 g ml^–1^). The cell suspension was overlaid on equal volume of HiSep and centrifuged at 400×g for 30 min with brakes off at 8°C. Following centrifugation, lymphocyte fraction at the interface between medium and HiSep was carefully aspirated, washed three times with PBS, counted and assessed for viability on a hemocytometer through trypan blue exclusion test. Viable cells (>95%) were adjusted to 2x10^6^ cells ml^–1^ in complete culture medium.

Basal as well as mitogen-induced *in vitro* lymphocyte proliferation was assessed using colorimetric assay based on tetrazolium salt (MTT) following the method of Berridge et al. (2005) [35]. Stock solution of mitogen was made in 0.2 M PBS (pH 7.2) at a concentration of 1 mg ml^–1^. Further dilution was made in culture medium. Flat bottom 96 well culture plates were used. To study basal or spontaneous proliferation, 50 µl splenic lymphocyte suspension was seeded into well of culture plate along with 150 µl of mitogen-free culture medium. Additional well contained only 200 µl of culture medium and served as blank. To study mitogen induced proliferation, 50 µl of different mitogens (Con A, PHA and LPS; final concentration was 10 µg ml^–1^)**,** 50 µl cell suspension (2x10^6^ cells ml^–1^) and 100 µl of culture medium (total volume 200 µl) were seeded into well of culture plate. To study effect of *in* *vitro* testosterone, 50 µl of different mitogens, 50 µl cell suspension (2x10^6^ cells ml^–1^) and 100 µl of culture medium containing different concentrations of testosterone (having final concentration of 10, 100 and 1000 ng ml^–1^) were added to each well. A set of wells that did not contain testosterone served as control. All samples were assayed in triplicates. Plates were incubated in humidified CO_2_ atmosphere at 25°C for 48 h. Following incubation, 20 µl of MTT reagent (5 mg ml^–1^) was added to each well, and plates were again incubated overnight in humidified CO_2_ atmosphere at 25°C. After incubation, the plates were centrifuged at 400×g for 10 min at 8°C. The supernatant was aspirated, and 100 µl of DMSO was added to each well to solubilize the formazan crystals. Absorbance was measured at 570 nm with the help of ELISA plate reader (Thermo Multiscan). Following blank subtraction, the triplicates are averaged.

### Statistical Analysis

Each experiment was repeated thrice. Data are presented as mean ± SEM. Means were compared by Analysis of Variance (ANOVA) followed by Newman Keuls multiple-range test.

## Result

**Figure 1:**
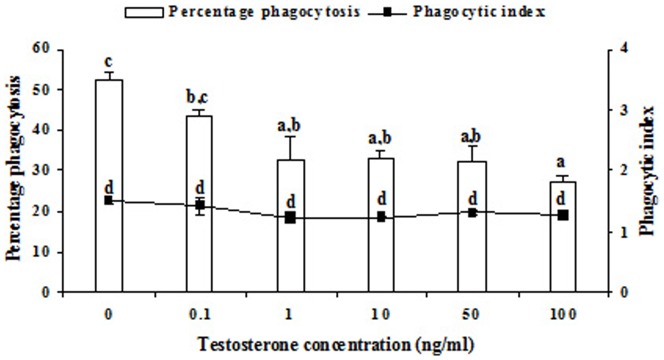
Effect of different concentrations of testosterone on splenic macrophage phagocytosis in the fresh water snake, *Natrix piscator*. The error bars bearing the same superscript do not differ significantly (Newman–Keul’s multiple-range test, p<0.05).

**Figure 2:**
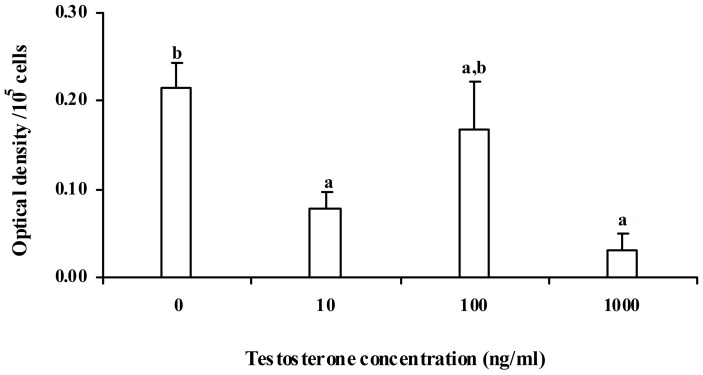
Effect of different concentrations of testosterone on NBT reduction in *Natrix piscator*. The error bars bearing the same superscript do not differ significantly (Newman–Keul’s multiple-range test, p<0.05).

**Figure 3:**
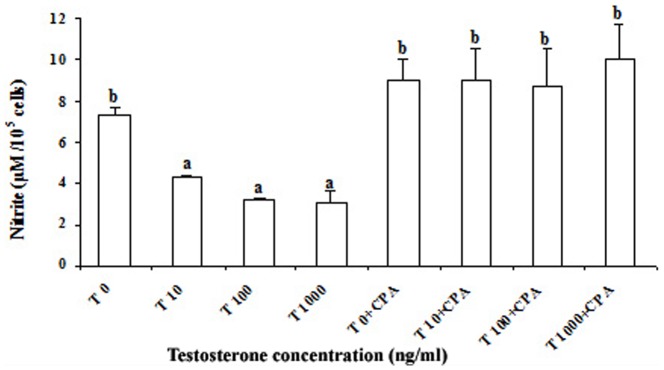
Effect of different concentrations of testosterone (T 10 to 1000– Testosterone 10 to 1000ng ml^−1^) on nitrite release in *Natrix piscator*. Effect of testosterone receptor antagonist cyproterone acetate (CPA, 100 ng ml^−1^) is also shown. The error bars bearing the same superscript do not differ significantly (Newman–Keul’s multiple-range test, p<0.05).

**Figure 4:**
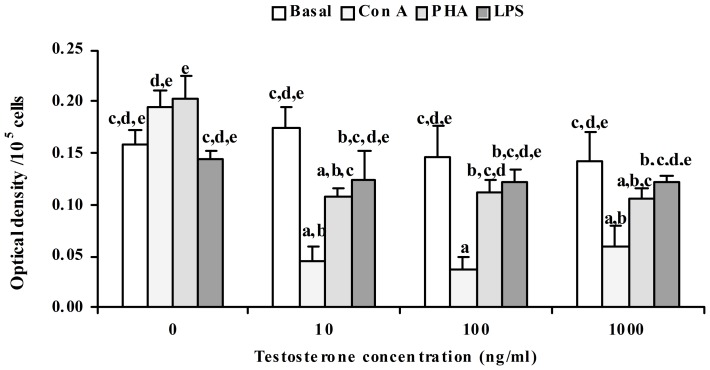
Effect of *in vitro* testosterone on mitogen induced splenocyte proliferation in *Natrix piscator*. Mitogens: ConA – Concanavalin A, 10 µg ml^–1^; PHA – Phytohemagglutinin 10 µg ml^–1^; LPS – Lipopolysaccharide, 20 µg ml^–1^). The error bars bearing the same superscript do not differ significantly (Newman–Keul’s multiple-range test, p<0.05).

*In vitro* testosterone treatment significantly (p<0.05) decreased the percentage phagocytosis by splenic macrophages, but, there was no change in phagocytic index (Fig. 1). Further, it was observed that decrease in percentage phagocytosis was significantly correlated with the *in vitro* testosterone concentration. Maximum reduction of percentage phagocytosis was obtained at 100 ng ml^–1^ concentration of testosterone. Our pilot experiment showed that testosterone below 10 ng ml^–1^ concentrations was not sufficient to influence NBT reduction and nitrite release. Hence, higher concentrations of testosterone, 10, 100 and 1000 ng ml^–1^ were used. Effect of *in vitro* testosterone on NBT reduction was found to be differential, as NBT reduction was decreased at 10 ng ml^–1^, but was unaffected at 100 ng ml^–1^, and again decreased at 1000 ng ml^–1^. The decrease was maximum at 1000 ng ml^−1^ testosterone concentration (Fig. 2). Nitrite release was significantly (p<0.05) reduced by *in vitro* testosterone treatment in a concentration dependent manner (Fig. 3). When the macrophages were pre-incubated with testosterone receptor antagonist, cyproterone acetate (CPA), the decrease in Nitrite release was alleviated, as Nitrite release was comparable to that of control splenocytes incubated in medium alone. There was no change in basal proliferation of splenic lymphocytes in relation to *in vitro* testosterone treatment; while Con A and PHA induced proliferative response reduced significantly (p<0.05) by *in vitro* testosterone. The reduction in Con A induced proliferative response was more apparent than that PHA induced one. However, there was no change in LPS induced proliferative response (Fig. 4).

## Discussion

The effect of gonadal steroids on lymphoid tissues is well known even before the importance of thymus in immune function had been recognized; researchers have noted thymic hypertrophy in response to gonadectomy [36]. The sex-related differences in immune capabilities are well established in mammals [37]. Females exhibit enhanced immunoreactivity compared to males as shown by higher immunoglobulin levels, better response to heteroantigens, and ability to combat infections [1], [14]. So far, the sex associated differences in immune parameters in reptiles are concerned, a few reports are available: *in vivo* administration of testosterone in either male or female lizard, *Chalcides ocellatus*, induces significant depletion of lymphoid elements, serum antibody titer to rat RBC and increase in skin allograft survival [38]. Decrease in the percentage of cortex, number of cortical and medullary lymphocytes in thymus and spleen of turtles, *Mauremys caspica*, have been found following testosterone administration [39], [40].

In the present investigation on cellular (splenocytes) immune responses, we found that the percentage phagocytosis was significantly decreased, when splenic macrophages were treated with testosterone, but no change occurred in the phagocytic index. Effect of testosterone was concentration dependent, and maximum reduction was obtained at 1000 ng ml^−1^ concentration. This finding corroborates to that of Mondal and Rai (1999, 2002) [3], [41], who reported that dihydrotestosterone had a suppressive effect, in a dose and duration dependent manner, on macrophage phagocytosis in wall lizard. Inhibition of macrophage phagocytosis was reported by *in vitro* testosterone in broiler chickens [42]. Implant of testosterone significantly reduced humoral immunity in both sexes of wild starling birds [43].

Reduction in nitrite release by snake splenic macrophages in a concentration dependent manner was found when treated with *in vitro* testosterone. Similarly, dose and time dependent sex steroids hormone regulation of nitrite release has been demonstrated by Mondal and Rai (2002) [41] in lizard’s splenic macrophages; but the results of present investigation differ from their study, as they have reported reduction at sex steroids concentrations ranging from 10^−4 ^ng ml^−1^ to 10^4^ ng ml^−1^; while we failed to find reduction in nitrite release at *in vitro* testosterone concentration below 10 ng ml^−1^: the reason might be that they have prestimulated macrophages by LPS before *in vitro* testosterone treatment.

In present study, effect of *in vitro* testosterone on NBT reduction was found to be differential, as NBT reduction was decreased at 10 ng ml^–1^ testosterone concentration, but was unaffected at 100 ng ml^–1^, and again decreased at 1000 ng ml^–1^. Such a biphasic effect of *in vivo* and *in vitro* estradiol on macrophage phagocytosis was reported in wall lizard [3]. The reduction of NBT is carried out by the superoxide anion (O_2_^–^) produced by granulocytes and macrophages. NBT reduction test is a measure of activation of oxidative burst, which has a high reactive microbicidal effect [44] in response to antigenic stimulation. NBT reduction test in absence of antigenic stimulation is also an indirect measure of the intracellular hexosemonophosphate shunt activity [45]. In the present study, it has been demonstrated that reptilian splenic macrophages seem able to produce an increase amount of O_2_^–^ and testosterone could modulate the metabolic pathway mentioned above.

The reports on sex steroids-induced modulation of macrophage cytotoxic function in mammals present a very confusing picture. Ovariectomy in mice diminished IL-1 secretion, whereas castration had no effect [25], [26]. The castration also failed to influence the *in vitro* TNF-α release by rodent macrophages [30]. On the contrary, the castration in male mice enhanced IL-1 and TNF- α secretion by peritoneal macrophages [46]. Similarly, gonadectomy irrespective of sex has been reported to increase the nitrite release by murine peritoneal macrophages suggesting that the sex hormone deprivation results in an increase in amount of nitrite release [28]. Savita and Rai (1998) [28] also reported that *in vitro* treatment of estradiol, testosterone and progesterone markedly reduced the nitrite release from LPS-activated macrophages in a dose and time-dependent manner in mice. In other studies in mammals, *in vitro* testosterone treatment reduces the phagocytic capacity of macrophages and inhibits nitrite release by macrophages [27], [47]. The present investigation suggests that testosterone modulates nitrite production via a receptor-mediated system, as treatment with testosterone receptor antagonist, cyproterone acetate (CPA), interfered with the action of testosterone and considerably reduced the testosterone-induced suppression of nitrite release of splenocytes though Benten et al. (2002) [48] have demonstrated a membrane testosterone receptor on lymphocyte, enabling a nongenomic response to testosterone in T-cells.

Reports on testosterone and lymphocytes proliferation in vertebrate species present a gloomy picture, as the deprivation of male sex steroid through castration either has no effect on lymphocytes proliferation [49] or decreased proliferation in hamster [50] or increased the splenocyte proliferation in rodent [15], [17] as well as circulating testosterone level is not related to splenocyte proliferation in voles [51]. Marco et al. (2009) [52] reported no significant effect of testosterone on lymphocyte proliferation or cytokine secretion in mice. Al-Afaleq and Homeida (1998) [42] reported *in vitro* testosterone caused inhibition of lymphocyte proliferation in Broiler chickens. In the present study, T-cell mitogen (Con A and PHA) and B-cell mitogen (LPS) induced splenocyte proliferation was studied in relation *in vitro* testosterone treatment. Basal proliferation did not change when different concentrations of testosterone were used. T-cell mitogen induced proliferation was suppressed by different concentrations of *in vitro* testosterone, and the suppression was more apparent in Con A induced proliferation. In contrast, B-cell mitogen (LPS) induced proliferation did not change significantly. The present results suggest that T and B cells of this reptilian species respond differently to the male sex steroid. Present finding reveals testosterone as inhibitory to splenocytes’ immune responses in *Natrix piscator*. Further, it may be suggested that by suppressing immune responses, testosterone may, therefore, act as a physiological mechanism regulating the relative amount of energy invested into either reproductive effort or immunocompetence.
